# Behavior change after alcohol intoxication in adolescents

**DOI:** 10.1186/1940-0640-8-S1-A81

**Published:** 2013-09-04

**Authors:** Mireille de Visser, Nico van der Lely, Eva van Zanten

**Affiliations:** 1 Department of Pediatric Psychology, Reinier de Graaf Group, Delft, the Netherlands

## 

To assess behavior change in (binge) drinking and its positive /risk factors in adolescents admitted with alcohol intoxication. Multicenter retrospective study screening for underlying psychosocial and cognitive problems with a structural interview and behavioral checklists at follow up 8 weeks after the event. All adolescents < 18 years old, admitted with a BAC > 0.05 g/L and reduced consciousness, seen in the years 2009 & 2010 were invited. Adolescents with medical co-morbidity, or immediate referral to Youth Social Services were excluded. A total of 355 patients were invited and 226 included in the analysis. In total 103 adolescents stopped drinking (45% male, average age 15.8 (15.5 - 16.0) years, average BAC 1.88 (1.78 - 1.98) g/L) and 106 continued drinking (57% male, age 16.1 (15.9 - 16.3) years, average BAC 2.00 (1.88 - 2.12) g/L). After multivariate analysis prior binge drinking (OR 5.287 (2.726 - 10.257), p = 0.000) and lack of negative consequences imposed by the parents after the event (OR 3.111 (1.594 - 6.071) p = 0.001) remained strongly associated with persistent drinking at follow up. Sub analysis of ongoing binge drinking identified age older than 16 years as a significant risk factor (OR 3.645 (1.314 - 10.114) p = 0.013). Strikingly, half of the adolescents admitted with alcohol intoxication do not learn from their negative experience. Binge drinking patterns and lack of negative consequences, are significantly associated with persistent alcohol use. Follow up should be directed towards patients and their parents with the aim to intervene and prevent ongoing alcohol use.

